# Mechanical Behavior of Lithium-Ion Battery Separators under Uniaxial and Biaxial Loading Conditions

**DOI:** 10.3390/polym16081174

**Published:** 2024-04-22

**Authors:** Sahand Shamchi, Behzad V. Farahani, Marian Bulla, Stefan Kolling

**Affiliations:** 1 Institute of Mechanics and Materials, Technische Hochschule Mittelhessen, Wiesenstr. 14, 35390 Giessen, Germany; bulla@altair.com (M.B.); stefan.kolling@me.thm.de (S.K.); 2 Soete Laboratory, EMSME Department, Faculty of Engineering and Architecture, Ghent University, 9052 Zwijnaarde, Belgium; behzad.vasheghanifarahani@ugent.be; 3 Altair Engineering GmbH, Josef-Lammerting-Allee 10, 50933 Cologne, Germany

**Keywords:** lithium-ion batteries, polymer separators, battery abuse tolerance, battery safety

## Abstract

The mechanical integrity of two commercially available lithium-ion battery separators was investigated under uniaxial and biaxial loading conditions. Two dry-processed microporous films with polypropylene (PP)/polyethylene (PE)/polypropylene (PP) compositions were studied: Celgard H2010 Trilayer and Celgard Q20S1HX Ceramic-Coated Trilayer. The uniaxial tests were carried out along the machine direction (MD), transverse direction (TD), and diagonal direction (DD). In order to generate a state of in-plane biaxial tension, a pneumatic bulge test setup was prioritized over the commonly performed punch test in an attempt to eliminate the effects of contact friction. The biaxial flow stress–strain behavior of the membranes was deduced via the Panknin–Kruglov method coupled with a 3D Digital Image Correlation (DIC) technique. The findings demonstrate a high degree of in-plane anisotropy in both membranes. The ceramic coating was found to negatively affect the mechanical performance of the trilayer microporous separator, compromising its strength and stretchability, while preserving its failure mode. Derived from experimentally calibrated constitutive models, a finite element model was developed using the explicit solver OpenRadioss. The numerical model was capable of predicting the biaxial deformation of the semicrystalline membranes up until failure, showing a fairly good correlation with the experimental observations.

## 1. Introduction

In response to rapidly evolving trends in communication and transportation industries, high-performance and low-cost energy storage solutions are required for a sustainable future. Lithium-ion (Li-ion) batteries offer many advantages such as high energy density, long cycle life, and low self-discharge; however, their safety under mechanical, thermal, and electrical abuse conditions is of great concern, c.f. [1,2,3,4,5]. This is particularly the case for plug-in hybrid electric vehicles (PHEVs) as well as electric vehicles (EVs), which have the potential of experiencing mechanical abuse loading.

One of the critical safety components within liquid electrolyte batteries is their separator. Its function is to prevent any physical contact between the positive (cathode) and negative (anode) electrodes while permitting free ionic transport [2,3]. The separator is required to be chemically and electrochemically stable while being mechanically strong to endure stresses caused during the battery’s assembly as well as throughout its operation (i.e., charging–discharging cycles exert a biaxial loading state on the separator) [4]. Any damage to its integrity would trigger an internal short circuit, which may lead to thermal runaway [6].

Battery separators can be categorized into three groups: microporous polymer membranes, non-woven fabric mats, and inorganic composite membranes [2]. The former, particularly the ones based on semi-crystalline polyolefin materials, have dominated the market for lithium-ion batteries owing to their good mechanical performance combined with high electrochemical stability, high porosity, and low cost [1]. These polyolefin-based microporous membranes can be manufactured either by dry processes or wet processes, which can substantially influence their mechanical behavior.

They usually come in the form of polyethylene (PE), polypropylene (PP), or their combination, namely, PE/PP (bilayer) [7] and PP/PE/PP (trilayer) [8,9]. The multilayer design offers an additional safety feature known as thermal shutdown; at a temperature below the occurrence of thermal runaway, the PE layer (with a melting point of 130 °C) turns into a nonporous film by melting and filling the pores, terminating the ionic flow and the battery operation, while the PP layer (165 °C) provides enough mechanical integrity to prevent a short circuit between the anode and cathode [3,5]. Due to this improved reliability, large industrial batteries have started utilizing trilayer separators. A study carried out by Li et al. [10] demonstrates the potential of multilayer membranes for use in high-safety lithium-ion batteries. Further information on monolayer separators and their benchmarking against multilayer ones can be found in [2,3,11,12,13].

Another issue with polyolefin-based separators is their low thermal stability which could lead to thermal runaway, raising safety concerns in the event of overheating. To enhance their electrochemical properties, separators are coated via inorganic particles such as alumina (Al_2_O_3_), silica (SiO_2_), titanium oxide (TiO_2_), and zirconia oxide (ZrO_2_) [14,15,16,17,18]. Gong et al. [15]. studied the influence of ceramic coatings on separators by coating an ultrathin layer of Al_2_O_3_ on a polyethylene (PE) separator via atomic layer deposition. The authors reported that ceramic deposition impedes separator shrinkage at elevated temperatures, enhances the thermal dimensional stability of the battery cell, and improves ionic conductivity and electrolyte wettability [15]. Apart from the safety concerns, the mechanical integrity of the separator also plays a substantial role in the electrochemical performance of Li-ion battery cells, compromising cell capacity and charge–discharge cycles; see [2]. 

In the literature, a number of research works investigating the mechanical behavior and failure mechanism of these microporous films exist [19,20,21,22,23,24]. Amongst them, Chen et al. [20] built a morphological model to illustrate the tensile deformation mechanism of dry-processed polymer separators in the machine direction (MD) and the transverse direction (TD). Using in situ tensile testing and atomic force microscope (AFM) imaging, the authors associated the observed anisotropic behavior of the dry-processed separator with its deformation mechanism: it stretched parallel to the MD results in the separation of crystalline lamellae along with the elongation of fibrils in the amorphous phase, followed by the breakage of the crystallite lamellae; loading along the TD, however, led to the breakage of the crystalline lamellae by chain pull-out; see [20]. 

Recent studies have focused on investigating the effects of compression stress on polypropylene separators in lithium-ion batteries. Hu et al. [25] developed a novel multiphysics model utilizing Thiessen polygons to simulate the structural evolution of a separator, correlating mechanical properties with battery performance. Xu et al. [26] employed a multiscale simulation methodology, reconstructing separator microstructures based on SEM images and conducting explicit dynamic simulations to analyze mechanical behavior and predict the performance of a Li-ion battery. Both studies highlight the effect of compression stress on reducing ionic conductivity, which in turn adversely affects the battery discharge capacity and increases cell temperature due the structural variation. These findings offer valuable insights for future efforts in optimizing separator design for enhanced battery performance and safety.

The biaxial mechanical behavior of monolayer PP (Celgard PP2075) and trilayer PP/PE/PP (Celgard 2325) separators was investigated by Kalnaus et al. [23]. Through employing Digital Image Correlation (DIC) technique, the authors reported a degree of sticking between the membranes and the surface of the spherical indenter; nonetheless, the final failure was in the form of a straight crack running along the MD. 

Zhang et al. [19] studied the mechanical integrity of dry-processed PE and trilayer (PP/PE/PP) separators under a uniaxial tensile condition (along the machine, transverse, and diagonal directions), compression, and biaxial punch tests. Therefore, two different failure modes under the biaxial loading condition were detected, either through the formation of a crack along the MD or one that exceeded beyond this stage and formed a transparent zone, followed by a zig-zag failure surface along the TD. The former is more prevalent and may increase the probability of a short circuit and the subsequent thermal reaction in a cell [19].

Concerning the rate sensitivity of microporous separators, there is a consensus in the literature: a positive strain rate dependency was noted under both tensile and compressive loadings owing to the viscoelastic behavior of separator materials, c.f. [27,28,29,30,31]. A number of studies thereby proposed a material model to describe the mechanical and fracture behavior of microporous separators; these include, among others, a linear viscoelastic model based on the Kelvin–Voigt model [28,32], a viscoelastic poroelastic model for a range of strain rates [33], an anisotropic crushable foam model [34], and a fully 3D microstructural model using the stochastic reconstruction approach [35].

The present study investigates the mechanical performance of two commercially available polyolefin trilayer (PP/PE/PP) separators under uniaxial and biaxial loading conditions. To generate a state of in-plane biaxial tension, a pneumatic bulge test setup was prioritized over the commonly performed punch test method. Derived from the experimentally calibrated constitutive models, a preliminary finite element model was developed to predict the biaxial deformation of the membranes. The accuracy of the acquired predictions was confirmed through a comparison with experimental observations.

## 2. Materials and Methods 

### 2.1. Separator Materials

Two types of microporous polymer separators were studied: Celgard^®^ H2010 (trilayer) and Celgard^®^ Q20S1HX (Ceramic-Coated Trilayer); both membranes encompass a PP/PE/PP composition with a thickness of 20 and 16 μm, respectively. The latter possesses an additional 4 μm ceramic coating on one side. The materials were supplied by Celgard (Celgard, LLC, Charlotte, NC, USA). Table 1 presents further information provided by the manufacturer. 

### 2.2. Uniaxial Tests 

Concerning the uniaxial loading configuration, the materials were tested in three directions, the machine direction (MD), transverse direction (TD), and diagonal direction (DD), conforming to the instructions proposed by ASTM D882 [36]. Samples were cut in strips dimensioned as 60 × 12 × 0.02 mm^3^ in length, width, and thickness, respectively. Generally, a width of 10 mm or more is recommended to enhance the reliability of the measurements and to minimize the deviation in the failure strain value [34].

Uniaxial tensile tests were carried out using a Hegewald and Peschke (Inspekt Table Blue) testing machine (Hegewald & Peschke Meß- und Prüftechnik GmbH, Nossen, Germany) with a 10 N load cell for the specimens in the TD and DD; a 100 N load cell, however, was employed for the MD specimens, owing to their higher strength. The values were registered at 5 Hz.

**Figure 1:**
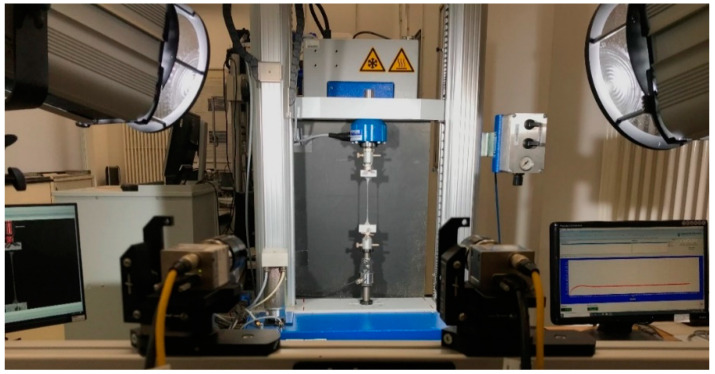
Uniaxial tension test setup equipped with the 3D-DIC system.

To prevent grip-induced failure and stress concentration, steel tabs, with a thickness of 0.1 mm, were introduced, resulting in a gauge length of 36 mm. Five tests per test arrangement were carried out under a constant crosshead speed of 0.1 mm/s, yielding a constant strain rate of 0.0027 s^−1^. All the experiments were carried out at room temperature. Figure 1 presents a general view of the uniaxial test setup.

### 2.3. Biaxial Tests

To model a more realistic loading condition which a separator may encounter during battery assembly and operation, biaxial tension experiments were carried out. They resembled a mechanical abuse condition that separators may endure under external impact loading or during battery charging–discharging cycles (by the expansion and shrinkage of electrodes in the battery cell) [2,19]. A state of in-plane biaxial tension on membranes is generally realized via a punch test configuration. Following ASTM F1306-90 [37], a small punch diameter (around 3.2 mm) was employed to assess the puncture resistance of separator films. However, biaxial stretching with larger hemispherical punch heads may restrain the material flow owing to the inevitable contact friction, thus affecting the strain distribution; see [19,23].

**Figure 2:**
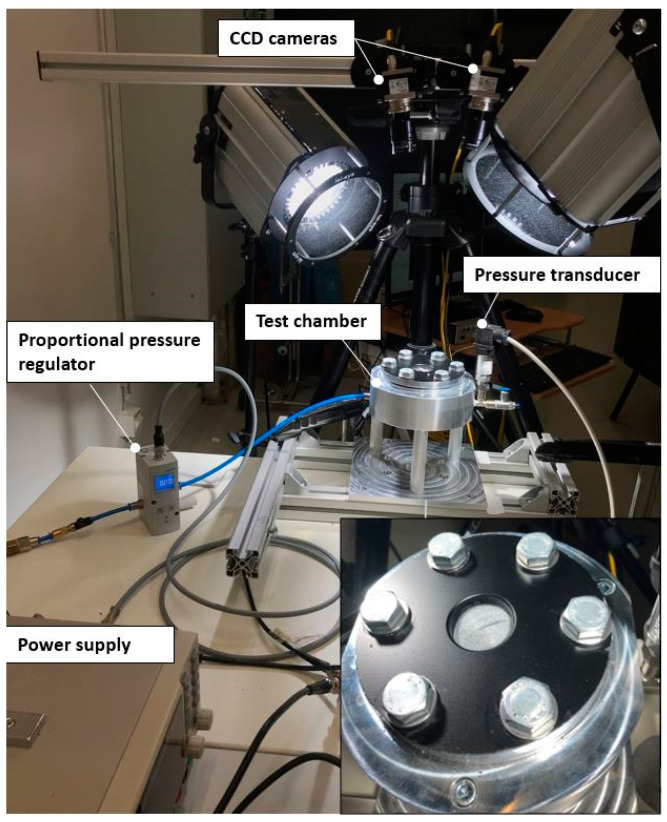
A view of the pneumatic bulge test setup.

To this end, a bulge test setup was built; a pneumatic system was prioritized over a hydraulic one owing to the sensitivity of polymers to moisture and their relatively low failure pressure. The setup, equipped with an optical measurement system, is shown in Figure 2. A test chamber was designed to enclose and inflate the membranes. The test specimen was sandwiched between two rubber sheets of 1 mm thickness. The assembly was clamped between the concentric upper and lower flanges, with an inner diameter of 32 mm, by applying a torque of 1.4 Nm. Thereby, a fixed boundary condition was attained without any slippage, air leakage, nor any grip-induced damage on the separator material.

A proportional pressure regulator VPPM, NPT (from Festo with a regulation range of up to 6 bar) was employed to provide a linear pressure increase in a controlled manner. The compressed air was directed to the test specimen through an air channel. The pressure history was registered using a 2.5 bar pressure transducer (Thermokon Sensortechnik GmbH, Mittenaar, Germany).

Regarding the data acquisition system, a measurement card, coupled with a PC equipped with DASYLab^®^ (2020) DAQ software, was employed. The software was also used to trigger the regulator. Prior to the main experiments, a series of preliminary tests were carried out with a polymer film to confirm the measurements and the test setup. Five tests per material were carried out. All the experiments were conducted at room temperature and under a pressure-controlled mode, with an average loading duration of 100 s, recorded at 20 Hz.

**Figure 3:**
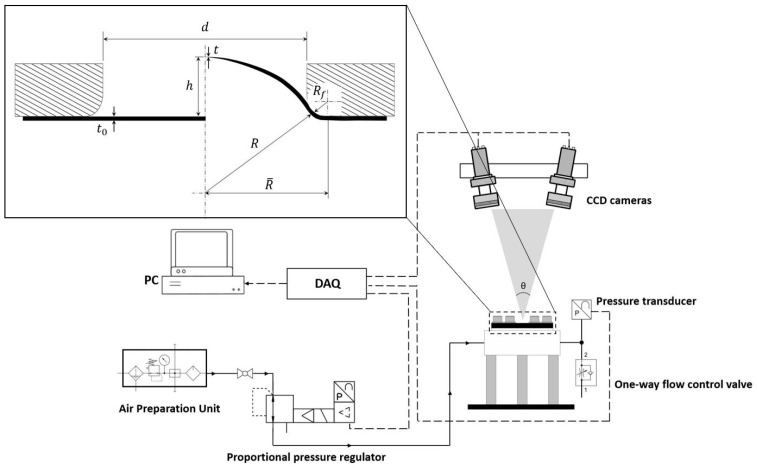
Schematic view of the pneumatic bulge setup with the geometrical parameters.

To attain the flow stress curves of biaxially deformed thin sheets, membrane theory is commonly employed. The theory is valid provided the ratio between the sheet thickness and the bulge diameter remains small, hence neglecting bending stresses; see [38,39]. Thereby, the biaxial stress can be obtained as follows:

(1) $$\sigma_{b}=\frac{pR}{2t}$$

where p, R, and t are, respectively, the pressure, curvature radius of the spherical dome, and the thickness of the membrane at the apex of the dome. Unlike the inflating pressure, the geometrical parameters can only be acquired indirectly; see Figure 3. As proposed by Hill [39], the curvature radius of the dome, assuming that is spherical, can be calculated analytically as follows:

(2) $$R=\frac{{\left(d/2\right)}^{2}+h^{2}}{2h}$$

where h and d are the dome height and the diameter of the bulge chamber. Panknin [40], as well as Shang and Shim [41], considered the effect of the fillet on the upper flange, $R_{f}$, ignored by Equation (2); therefore, the radius of the dome can be defined as follows:

(3) $$R=\frac{{\bar{R}}^{2}+h^{2}}{2h}-R_{f}$$

As the membrane is inflated, the meridional profile of the membrane, at this interface, is assumed to be tangential to the round edge of the flange. Equation (3) solely requires the continuous registration of the dome height and remains valid up to h/d=0.28, c.f. [38,40].

With respect to the quantification of the thickness value at the dome apex, a pragmatic model, as a function of curvature radius, was proposed by Kruglov et al. [42]. Thereby, assuming the incompressibility of the material, the instantaneous thickness at the pole position can be expressed as [42] follows:

(4) $$t=t_{0}{\left[\frac{\left(d/2\right)/R}{arcsin\;\left(\left(d/2\right)/R\right)}\right]}^{2}$$

The thickness of the undeformed membrane is designated as $t_{0}$. There exists a number of other approaches to quantify the instantaneous thickness and bulge radius [43,44]; nonetheless, the methodologies proposed by Panknin [40] and Kruglov et al. [42] were incorporated in this study, as their combination, proven by [43], yields a more accurate determination of the flow stress curve.

### 2.4. Optical Measurement

The experimental setups were coupled with a stereovision 3D Digital Image Correlation (DIC) system—a non-interferometric technique that employs image-matching algorithms to quantify the displacement and strain values of an object. 

For both setups, the images were recorded using two Basler ace cameras (12 MP resolution) with a CMOS image sensor and illuminated by two spotlights. The cameras were equipped with Xenoplan 2.8/50 lenses (Jos. Schneider Optische Werke GmbH, Bad Kreuznach, Germany). The working distance and acquisition interval were accordingly set to 920 mm and 4 fps (for uniaxial tests) and 450 mm and 5 fps (for biaxial experiments). With the given optical measurement system, pixel sizes of approximately 17.2 px/mm and 50.9 px/mm were attained, respectively, for the uniaxial and biaxial experiments. However, in the case of uniaxial tests in the diagonal direction, the working distance of 1250 mm (pixel size of 12.8 px/mm) was selected in order to accommodate larger strain values of around 350%, thereby circumventing the usage of crosshead displacement.

To enhance the image correlation, a random speckle pattern was applied to the specimens. As for the postprocessing, commercial software, VIC-3D (Version 8), from Correlated Solutions©, was employed. A subset size of 29 pixels was used for image analysis. The usage of 3D-DIC enabled us to quantify the dome height and strain values, proving to be an indispensable complement, particularly to the bulge test.

## 3. Results and Discussion

This section is dedicated to presenting the results obtained from experiments documented by the DIC. Subsequently, a detailed discussion of the results is provided to offer readers comprehensive insights. Furthermore, a preliminary numerical analysis is conducted to validate the pressure variation concerning displacement for both studied membranes.

### 3.1. Uniaxial Tests

**Figure 4:**
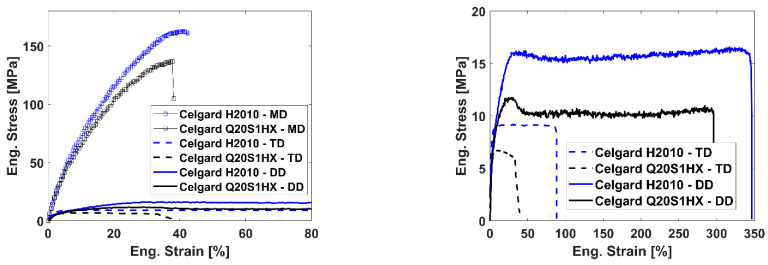
Representative engineering stress–strain response of both membranes under uniaxial loading condition.

Uniaxial tensile tests have been performed according to the test configuration described in the previous section. As a result, Figure 4 presents the representative stress–strain curves of both Celgard membranes under uniaxial loading condition. As anticipated from dry-processed polymer-based separators, both membranes demonstrate a high degree of in-plane anisotropy. 

For the MD-oriented samples, a mean strength of 164.5 ± 5 MPa and 131.8 ± 7.2 MPa, with a coefficient of variation of 3% and 5.4%, were measured, respectively, for Celgard H2010 Trilayer and Celgard Q20S1HX Ceramic-Coated Trilayer. However, the strength values attained in the transverse and diagonal directions, for both membranes, were lower than that of the MD by an order of magnitude; see Figure 4.

This orientation dependence stems from the deformation mechanism (breakage and separation) of crystallite lamellae, arranged parallel to the transverse direction, along with the generation and elongation of pores parallel to the machine direction (i.e., these MD-oriented oval-shaped pores impede the propagation of the crack, hence increasing the failure strength) [19,20].

The TD and DD-oriented samples demonstrated bilinear behavior with rather evident yield points, followed by a plateau under a relatively stable stress state until rupture. However, in the DD case, both separators tolerated much higher strain values, reaching a mean failure strain of 318% and 290%, sporadic to a degree, with a coefficient of variation of 22.5% and 27%, respectively, for Celgard H2010 and Celgard Q20S1HX. Such a relatively large variation with the dry-processed separators on the DD was also reported by [34]. It is worth mentioning that only Celgard Q20S1HX underwent strain softening, particularly in the diagonal direction by a decline of around 13%, which proceeded by a cold drawing region, i.e., the plateau.

**Figure 5:**
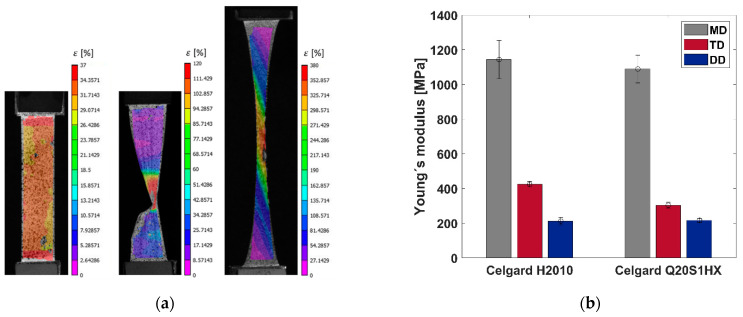
(**a**) The strain distribution of Celgard Q20S1HX stretched along MD, TD, and DD (left to right); (**b**) the overall comparison of the Young’s moduli for both membranes.

Figure 5a shows the full-field strain map of Celgard Q20S1HX, stretched in three directions, prior to the failure (the DD-oriented sample at a strain value of around 120%). When stretched along the MD, a homogenous strain distribution was observed in all the samples up until rupture, with failure gravitating towards the gripping zones. With the TD- and DD-oriented samples, however, strain localized within a shear band parallel to the machine direction, resulting in a fairly straight failure surface, as opposed to the wrinkling effect observed with the MD samples. Such pronounced strain accumulation in bands arises from the elongation of crystallite lamellae (i.e., the bulk section parallel to the transverse direction) and the pores in the membrane. The failure mechanism of microporous polymer separators is elucidated in other related works, c.f. [2,19,20].

A comparable trend in the strain evolution for Celgard H2010 was detected, which is not included here for the sake of brevity. Figure 5b shows the measured Young’s moduli for both membranes.

The mechanical behavior of membranes in the machine direction can be described by Swift’s law [45], the power law as shown in Equation (5), and Voce’s law [46], the exponential law as shown in Equation (6), formulated below.

(5) $${\bar{\sigma }\left(\bar{\varepsilon_{p}}\right)}_{Swift}=A{\left(\bar{\varepsilon_{p}}+\varepsilon_{0}\right)}^{n}$$

(6) $${\bar{\sigma }\left(\bar{\varepsilon_{p}}\right)}_{Voce}=\sigma_{0}+Q\left(1-e^{-\beta {\bar{\varepsilon }}_{p}}\right)$$

where the following terms are used:

$\bar{\varepsilon_{p}}$—Equivalent plastic strain;
*A*—Swift hardening coefficient (MPa);
$\varepsilon_{0}$—Swift hardening parameter;
*n*—Swift hardening exponent;
$\sigma_{0}$—Voce hardening parameter (MPa);
*Q*—Voce hardening coefficient (MPa);
*B*—Voce plastic strain coefficient.

Table 2 reports the hardening parameters associated with Swift’s and Voce’s laws characterized for both H2010 and Q20S1HX membranes in the MD.

Moreover, G’Sell–Jonas [47,48] proposed a constitutive model to assess the behavior of semicrystalline polymers by incorporating both viscoelasticity and viscoplasticity in a material model. Therefore, the flow behavior under a constant strain rate at room temperature can take the following formulation shown in Equation (7). Notice that the recent equation is a modified equation as presented in [29], in which

(7) $$\bar{\sigma }\left(\bar{\varepsilon_{p}}\right)=K+B\left(1-e^{-C{\bar{\varepsilon }}_{p}}\right).\left(1+D{\bar{\varepsilon }}_{p}+F{{\bar{\varepsilon }}_{p}}^{2}\right)$$

where the following terms are used:

$\bar{\varepsilon_{p}}$—Equivalent plastic strain;
*K*—Initial yield stress parameter (MPa);
*B*—Hardening coefficient (MPa);
*C*—Hardening plastic strain coefficient;
*D*—Second hardening plastic strain coefficient;
*F*—Third hardening plastic strain coefficient.

**Figure 6:**
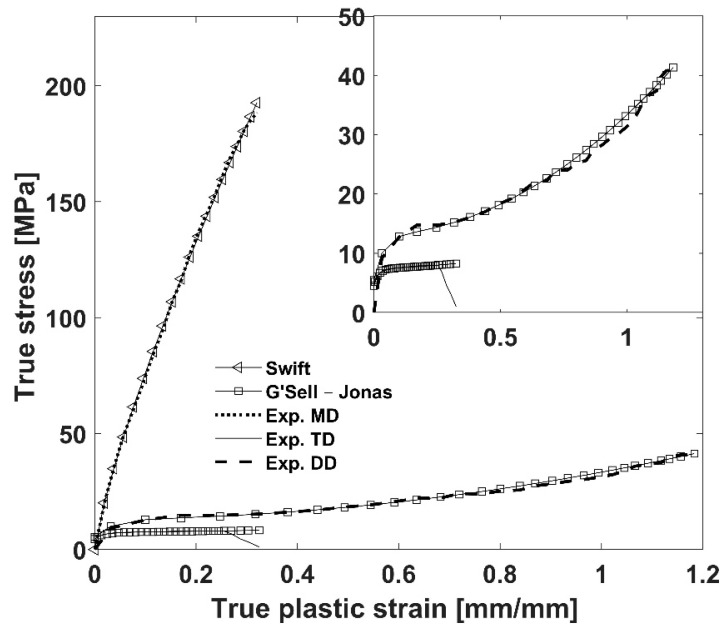
Representative fitted hardening curves against the experimental data for Celgard Q20S1HX.

In addition, Table 3 presents material coefficients for the G’Sell–Jonas model achieved for both the H2010 and Q20S1HX membranes in the TD and DD. Figure 6 illustrates the true stress in terms of true plastic strain curves derived from the Swift and G’Sell–Jonas models.

It is noteworthy that the principal material coefficients of the G’Sell-Jonas model have been experimentally documented in this study, which is invaluable for prospective applications. Although the model was originally designed to accommodate strain rate dependency, for the purposes of this study, strain dependency has been disregarded in the numerical modeling study.

### 3.2. Biaxial Tests

**Figure 7:**
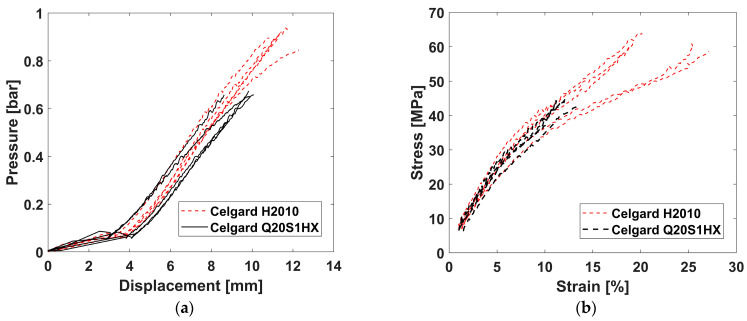
(**a**) The output from the pneumatic bulge test; (**b**) the biaxial stress–strain response of separators.

Figure 7a,b present the response of both separator films under a biaxial loading state. Overall, the findings demonstrated good repeatability, and mean critical principal strain values of 22% ± 3.7 and 11.8% ± 1.1 were attained, respectively, for the H2010 and Q20S1HX membranes. Likewise, the required failure pressure for the PP/PE/PP trilayer (Celgard H2010) was around 0.9 bar, while the ceramic coating of a similar membrane (Celgard Q20S1HX) resulted in a decline of 29%. 

A similar trend was detected with the uniaxial tests; H2010 outperformed Q20S1HX, demonstrating higher strength and stretchability. Nonetheless, despite a degree of compromise on the mechanical performance of Q20S1HX, applying a thin layer of ceramic particles on the polyolefin-based membranes enhanced the thermal stability and wettability of an otherwise hydrophobic film—a positive contribution to the overall safety of the battery [5,18].

**Figure 8:**
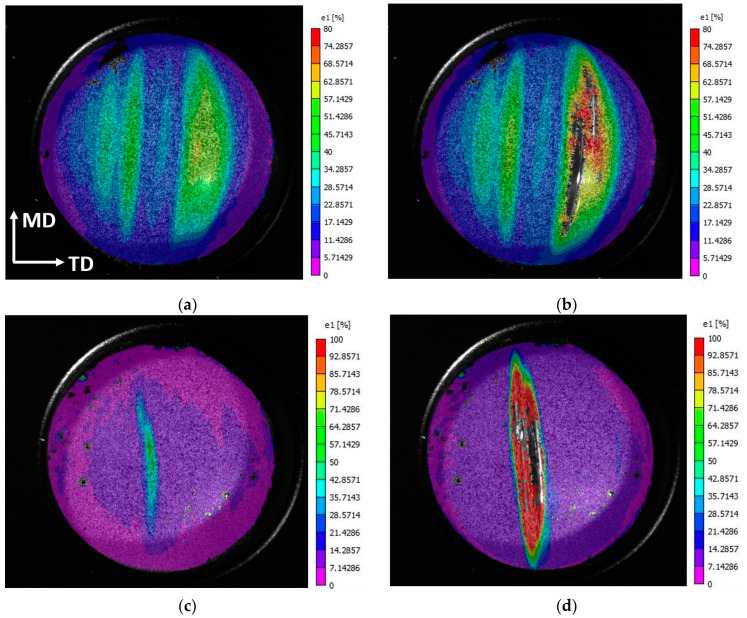
The principal strain distribution under in-plane biaxial tension in (**a**,**b**) Celgard H2010 and (**c**,**d**) Q20S1HX.

The principal strain distribution of Celgard H2010 and Celgard Q20S1HX is presented in Figure 8. The use of a pneumatic system enabled a free flow of the material; this may not be the case with a punch test, as the membrane may partially stick to the hemispherical indenter as it stretches, as demonstrated in [19,23], rendering a more biased deformation state. 

Both membranes reveal a similar failure mode through the formation of a crack running along the machine direction, owing to the highly anisotropic nature of the dry-processed separators. In a number of tests, the strain was accumulated along a small number of bands always parallel to each other, mostly passing with an offset from the dome’s center line. The failure surface, however, was smooth, similar to the one observed under uniaxial tension loading along the TD (see Figure 5a), which arose from the elongation of crystallite lamellae in the membrane. 

It is also worth noting that the failure was preceded by the formation of transparent bands. This is evident from the DIC footage captured prior to the failure, as shown in Figure 8b,d. However, Figure 8a,c illustrate the onset of transparent zones, which, on average, corresponded to strain values of around 17.5% (for H2010) and 7.6% (for Q20S1HX), as presented in Figure 7b. With the final failure values in mind, Celgard H2010 demonstrated a favorable performance with delayed localization, as confirmed via the 3D-DIC analysis. However, the start of thinning and the formation of a transparent zone may trigger an internal short circuit, altering the electrochemical performance of a battery cell prior to the actual failure of the separator.

### 3.3. Finite Element Analysis

**Figure 9:**
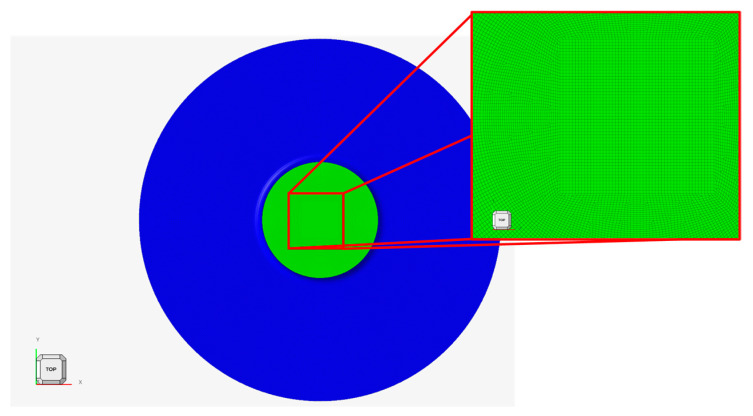
Top view of the numerical model: the separator is in green, while the upper holder is in blue; lower holder disc with the magnification on the FE mesh.

This section deals with the numerical modeling simulation on the biaxial loading condition through finite element method (FEM) formulations. Here, an explicit FEM model was generated using commercial software Altair Engineering Inc. (Troy, MI, USA) HyperWorks 2022.2. Figure 9 shows a general view of the numerical model with details on the FE mesh. The FE mesh was formed using four-node quadrilateral elements with an element size of 0.2 mm.

**Figure 10:**
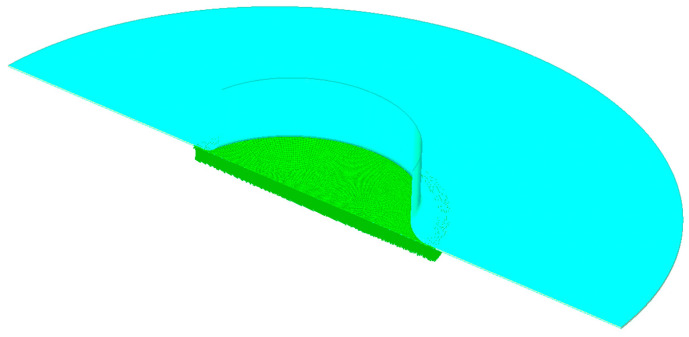
Iso- and section-cut view of the model, showing the area of applied pressure.

Given the fact that the current study did not cover the strain rate dependency of the investigated materials, the applied material hardening curve uses only the quasi-static hardening curve. Regarding the boundary conditions, the pressure rate ranged between 0 and 100 bar within 1 s in which the pressure load was applied on the central area of the separator material, perpendicular to the surface; see Figure 10. The constitutive law incorporating elastoplastic characteristics was employed. This law was specifically associated with Hill’s yield surface, a mathematical representation defining the onset of plastic deformation in materials. Notably, this constitutive law underwent a distinct calibration process, with a particular focus on accurately characterizing and fine-tuning its parameters within the plastic region of the material’s stress–strain behavior.

Regarding the material failure model, a tabulated hardening curve was considered with a Hill-based material model, as used in a previously published paper [24]. It aimed to achieve the calibration and validation of the orthotropic failure model proposed by Bulla et al. [24]. Therefore, Table 4 and Table 5 accordingly report the fracture strain limits for Celgard H2010 and Celgard Q20S1HX for all three directions. The presented values have been derived from the performed experiments in 0°, 45°, and 90° orientations and adjusted to the element size. No strain-rate-dependent values were used within this failure model.

Simulations were performed using the explicit FEA solver OpenRadioss (Altair Engineering Inc., Troy, MI, USA) on a Windows64 computer with four Intel i7-6820 CPUs at 2.7 GHz with 64GB RAM. OpenRadioss solved the entire simulation with 230,000 cycles and an average timestep of 7 × 10^−5^ to 1 × 10^−5^ ms. The simulation duration for 42 ms of simulation time was about 11 h and 6 min, with a total number of 1,096,301 cycles. 

**Figure 11:**
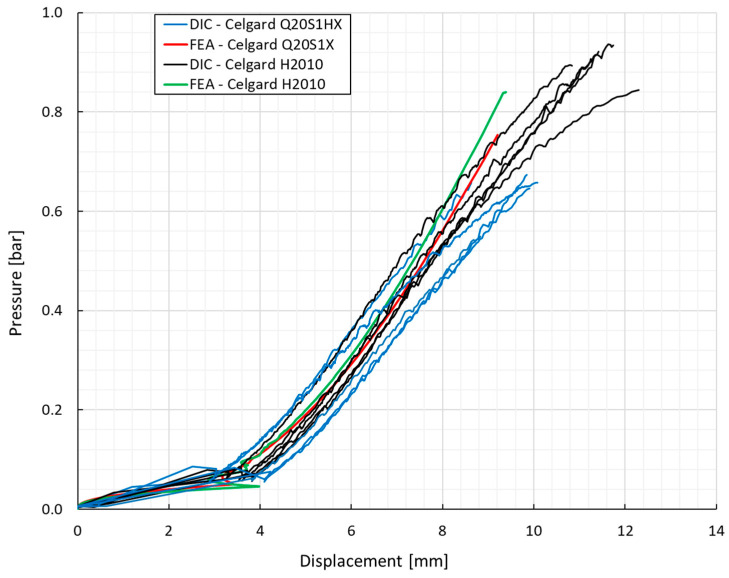
Pressure vs. displacement response of separator materials under biaxially loaded condition. Experimental and numerical results.

Figure 11 shows the pressure in terms of displacement variations obtained for both studied membranes subjected to the biaxial loading condition. With a closer look at the results, it can be inferred that Celgard H2010 possesses higher stiffness/rigidity at the corresponding displacement, exposing higher ductility. In other words, this material would have better capability to resist deformation under the applied pressure, leading to more resistance to change in shape. This phenomenon has already been proved by the Young’s modulus characterized through the DIC analysis.

Concerning the material instability which plays a key role in orthotropic material models, it must be mentioned that as the elements in the material became closer to the critical strain levels, the material underwent instability, leading to localized deformation within these elements. On the other hand, other elements began to endure relaxation. This meant that the total deformation was uniformly distributed across numerous elements in the initial state. Nevertheless, as the material experienced plastic behavior, the element size became a key factor in determining fracture behavior. Owing to the aforementioned considerations, the element size has been properly selected in this FEA, in which the material did not experience significant instability. 

In addition, four-node quadrilateral elements reflected a proper performance to simulate the nonlinear behavior of the studied materials. Thin structures often involve large deformations, and the elements used should be less sensitive to mesh distortions. Quadrilateral elements generally exhibit reduced sensitivity to mesh distortions compared to triangular elements, providing better numerical stability. 

Overall, the numerical model was able to capture the pressure variation versus the displacement of the membranes subjected to the biaxial loading condition, yielding a fairly good correlation with the experimental observations.

## 4. Conclusions

The mechanical integrity of two polyolefin trilayer (PP/PE/PP) separators was studied. Both separators displaced a notable anisotropy. Celgard H2010 exhibited superior mechanical performance compared to Celgard Q20S1HX, demonstrating higher strength and stretchability under both biaxial and uniaxial loading conditions. The resilience of Celgard H2010 mitigates the risk of internal short circuits between electrodes, thereby improving battery safety and longevity. 

While surface coating polyolefin separators with ceramic material (Celgard Q20S1HX) provides the benefits of enhanced wettability and thermal stability, it appears to compromise, to a degree, the mechanical performance. However, both separators demonstrated a comparable failure mode in the form of thinning and semi-transparent zones running along the machine direction, as confirmed via DIC analysis. 

Lastly, a preliminary finite element model of the biaxial tests was developed in OpenRadioss. The numerical model was able to capture the pressure–displacement behavior of the separators fairly well concerning the experimental findings. Advanced numerical modeling is ongoing to better simulate the failure behavior of the studied materials. 

## Figures and Tables

**Table 1:** Properties of the membranes.

Product Name	Composition	Thickness [μm]	Porosity [%]	Gurley (JIS) [Sec]	Manufacturing Process
Celgard H2010 Trilayer Membrane	PP/PE/PP	20	46	240	Dry-processed
Celgard Q20S1HX Ceramic-Coated Trilayer Membrane	PP/PE/PP	20	49	195	Dry-processed

**Table 2:** Swift and Voce hardening parameters for the machine direction.

	*A* [MPa]	*ε*_0_	*n*	*σ*_0_ [MPa]	*Q* [MPa]	*B*
H2010	613.12	0.9	0.82	2.81	522.0	1.67
Q20S1HX	554.40	0.83	0.79	3.87	396.3	1.98

**Table 3:** The material coefficients for G’Sell–Jonas model.

	*K* [MPa]	*B* [MPa]	*C*	*D*	*F*
H2010–TD	3.46	5.556	285.96	2.24	0.314
Q20S1HX–TD	4.52	2.780	61.88	0.83	0.743
H2010–DD	6.51	13.06	14.84	0.14	2.76
Q20S1HX–DD	5.45	7.54	29.12	0.096	2.60

**Table 4:** Fracture strain limits used within the failure criterion for Celgard H2010.

	Uniaxial Compression	Pure Shear	Uniaxial Tension	Plain Strain Tension	Biaxial Tension
0°	1.20	0.32	0.12	0.072	0.084
45°	33.0	8.91	3.30	1.98	0.084
90°	11.0	2.97	1.10	0.66	0.084

**Table 5:** Fracture strain limits used within the failure criterion for Celgard Q20S1HX.

	Uniaxial Compression	Pure Shear	Uniaxial Tension	Plain Strain Tension	Biaxial Tension
0°	1.3	0.351	0.13	0.08	0.091
45°	35.0	9.450	3.50	2.10	0.091
90°	14.5	3.915	1.45	0.87	0.091

## Data Availability

The original contributions presented in the study are included in the article, further inquiries can be directed to the corresponding author.
